# Aspirin Use and Lung Cancer Risk: A Possible Relationship? Evidence from an Updated Meta-Analysis

**DOI:** 10.1371/journal.pone.0122962

**Published:** 2015-04-07

**Authors:** Hai-yan Jiang, Tian-bao Huang, Lei Xu, Jing Yu, Yan Wu, Jiang Geng, Xu-dong Yao

**Affiliations:** 1 Department of Urology, Shanghai Tenth People's Hospital, Tongji University, Shanghai, China; 2 Electrocardiographic room, Shanghai Tenth People’s Hospital, Tongji University, Shanghai, China; 3 Department of First Clinical Medical College, Nanjing Medical University, Nanjing, Jiangsu, China; 4 Department of respiratory medicine, Shanghai Tenth People's Hospital, Tongji University, Shanghai, China; 5 Department of Ophthalmology, Shanghai Tenth People's Hospital, Tongji University, Shanghai, China; University of Birmingham, UNITED KINGDOM

## Abstract

**Background and Purpose:**

Growing evidence has emerged and controversial results reported on possible relationship between aspirin use and lung cancer risk. We, therefore, conducted this updated and comprehensive meta-analysis to evaluate this issue, with focus on dose-risk and duration-risk relationships.

**Methods:**

We searched electronic databases including PUBMED, EMBASE and Cochrane library to identify eligible studies. Relative risk (RR) and its 95% confidence interval (CI) were used for cohort studies, while odds ratio (OR) were employed for case-control studies. The random effects and fixed effects models were used for analyses.

**Results:**

18 studies were identified including 19835 lung cancer cases, which were eligible for inclusion in the present meta-analysis. Pooled data from case-control studies showed a significant inverse association between regular aspirin use and lung cancer risk. But for cohort studies, insignificant association was detected with little evidence of heterogeneity (RR: 1.05, 95%CI: 0.95 – 1.16; I^2^: 10.3%, p value: 0.351). In case-control studies, standard aspirin use (>325mg) was related to lower lung cancer incidence, compared with low-dose aspirin use (75–100mg). A similar trend was observed in cohort studies. Besides, when analysis was restricted to long time regular aspirin use (>5 years), insignificant results were reported in both cohort and case-control studies. Finally, regular aspirin use might result in higher reduction of non-small cell lung cancer incidence among men.

**Conclusions:**

Our findings do not support the protective effect of regular aspirin use on lung cancer risk. Long time aspirin use, sex, dose and type of lung cancer might alter the effect of aspirin use on lung cancer risk. More well-designed studies are needed to further clarify these associations.

## Introduction

Lung cancer is the most commonly diagnosed cancer with 1.61 million new cases in 2008, representing 12.7% of all cancer cases[1]. In addition, it is the first cause of cancer death with 1.38 million deaths worldwide[2]. Considering the low five-year survival rate, identification of effective methods for chemoprevention is of prime importance.

Aspirin, one of the most commonly used non-steroidal anti-inflammatory drugs (NSAIDs), was shown to have protective effects on colorectal adenoma by inhibiting COX-2 enzymes, restoring normal cell apoptosis and reducing angiogenesis[3]. In addition, growing evidence has emerged that aspirin use might reduce the incidence and mortality in various cancers, including colorectal[4], esophageal[5] and gastric [6]cancers. However, controversial associations between aspirin use and lung cancer risk have been reported in epidemiologic studies [7–10]. Recently, several large-scale well-designed studies were published. McCormack et al.[11]synthesized eight observational studies of International lung cancer consortium (ILCCO) and reported a strong inverse association in males (pooled RR = 0.73, 95%CI: 0.57–0.92), but opposite results were obtained for women (pooled RR = 1.02, 95%CI: 0.87 to 1.19). Similar results were reported by Cook et al.[12]. 100 mg of alternate-day aspirin might not reduce lung cancer risk in healthy women, after more than ten years of follow-up. As for mortality, Brasky et al. [13] summarized the relevant data in Vitamins and Lifestyle Cohort and found that relative to non-use, neither high (≥4 days/week and ≥4years) pre-diagnostic use of regular standard nor low-dose aspirin did not reduce lung cancer death (HR 0.99, 95% CI: 0.74 to 1.33 and HR: 0.89, 95% CI: 0.67 to 1.17, respectively). However, another population-based cohort study performed by Jonsson et al. [14] indicated that low-dose aspirin use was associated with lower tumor extent in lung cancer (p<0.0001) and lower rate of metastatic disease (OR = 0.8, 95%CI: 0.7–0.9), suggesting that mortality of lung cancer may be decreased by low-dose aspirin use.

A previous meta-analysis conducted by Xu et al.[15] revealed an inverse association between aspirin use at a dose of 7 tablets per week and lung cancer risk (pooled OR = 0.80, 95%CI: 0.67 to 0.95). Both of another two meta-analyses [16, 17] did not find any association by combining cohort studies and case-control studies. Significant differences were shown among these two study types. Besides, the possible relationships between regular standard aspirin use or low-dose aspirin use and lung cancer risk are still unclear. To date, several another studies [12, 18] published and reported different results. Considering the different levels of evidence, we, therefore, conducted this up-to-date and comprehensive meta-analysis to evaluate the effect of regular aspirin use on lung cancer risk by separating cohort studies, case-control studies and randomized controlled trials (RCTs), focusing on dose-risk and duration-risk relationships. Meanwhile, the influences of gender and cancer pathological type on these associations were also assessed.

## Methods

### Data Source and Search Strategy

PUBMED, EMBASE and Cochrane library were searched between January 1966 and December 2013 to identify eligible studies, using the following key words: ‘aspirin or acetylsalicylic acid or non-steroidal anti-inflammatory agent or NSAID’, ‘lung cancer’ and ‘risk’. Besides, the reference list of the articles and reviews retrieved were manually searched to identify additional eligible studies.

### Criteria for Inclusion and Exclusion

Studies were eligible for inclusion if they: 1) were case-control or cohort studies, or RCT; 2) evaluated the influence of aspirin use on lung cancer risk separately from other NSAIDs; 3) had explicit description of aspirin exposure and 4) provided relative ratios (RRs) or odds ratios (ORs) with 95%confidence intervals (CIs) or sufficient information to calculate them. Review articles, case reports, letters to the editor and editor comments were excluded. If data were duplicated in more than one study, the most recent or comprehensive report was selected for analysis.

### Data Extraction

Data extraction was carried out independently by two investigators (Hai-yan Jiang and Tian-bao Huang) according to the meta-analysis of observation studies in epidemiology guidelines [19], and discrepancies were adjudicated by Xu-dong Yao. The data extracted from each study included first author name, year of publication, gender, country, study design, type of controls, sample size, definition of aspirin exposure, RRs or ORs and their 95%CIs and adjusted factors. Data on standard and low-dose regular aspirin use and relevant incidence of lung cancer were also extracted. Moreover, if data were available, lung cancer cases were subdivided into small cell lung cancer (SCLC) and non-small cell lung cancer (NSCLC). When multiple estimates were reported, the one adjusted for the most variables was selected.

### Statistical Analysis

RRs and their 95% CIs were used to assess the data extracted from cohort studies, while odds ratios (ORs) were employed for case-control studies. Considering the recall bias and selective bias of case-control studies, we did not pool data from RCT, cohort studies and case-control trials. When data of different durations of use or different intake levels were available, we subsequently restricted the analyses to regular standard aspirin use or low dose aspirin use with longest duration. Regular standard aspirin use was defined as “regular aspirin use with dose ≥325mg”, while low-dose aspirin use was considered for “regular aspirin use with dose between 75 to 100mg”. Long time regular aspirin use was defined as “regular aspirin use for more than 5 years, regardless of the dose”. The statistical heterogeneity among studies was evaluated by using the Cochrane’s Q and I^2^ statistics. As for Q statistics, heterogeneity was considered for P<0.05. When P>0.05 and I^2^<50%, the studies were considered with acceptable heterogeneity, and the fixed effects model was used. Otherwise, the random effects model was used.

Subgroup analyses based on gender (female only vs. male only vs. both sex) and lung cancer histology (NSCLC vs. SCLC, adenocarcinoma vs. squamous cell carcinoma) were carried out to detect the source of heterogeneity. Smoking is well known as one of the most important risk factors for lung cancer. Therefore, sensitivity analyses, excluding studies which did not adjust for smoking, were conducted to minimize the influence of smoking on lung cancer risk.

Finally, potential publication bias was evaluated graphically with funnel plots of log risk ratio against the standard error of the study. If the funnel plot was asymmetrical, the rank correlation method proposed by Begg et al. and linear regression approach suggested by Egger et al. were used to evaluate the potential publication bias. At P < 0.05, sensitivity analyses were conducted to explore whether the final effects were strongly influenced by individual studies. All statistical analyses were performed by using the STATA Statistical Software version 11.1 (Stata Corp., College Station, Texas, USA), and two-tailed P values were determined.

## Results

**Fig 1:**
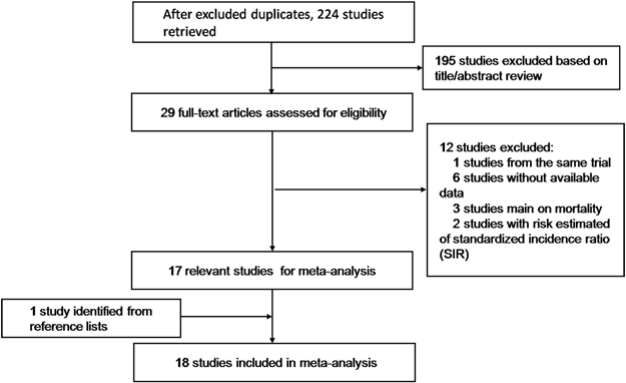
The detailed steps of literature search in the present meta-analysis.

The detailed flow diagram of the literature search is displayed in Fig 1. Briefly, 18 observational studies were identified involving 19835 incident cases of lung cancer, and were eligible for inclusion in the present meta-analysis. They included seven case-control studies[10, 18, 20–24]assessing 5833cases and 11210 controls, nine cohort studies[7–9, 25–30]evaluating 9262 cases among 450812 subjects, one post-trial of RCT[12]analyzing 431 cases among 39876 subjects, and a large-scale multicenter study[11] directed by ILCCO. McCormack et al.[11]pooled eight ILCCO studies that had data on aspirin or NSAID use prior to diagnosis, including one cohort-and seven case-control studies. Considering the level of evidence, the latter was assigned to the case-control group. As for gender, nine [8, 11, 12, 18, 20, 24, 27, 28, 30] of the included studies were carried out in female populations, four [9, 11, 24, 30]were in males and the remaining five dealing with both males and females. Of the 18 studies, eight [9, 12, 20, 22–24, 27, 28] referred to histology type of lung cancer and eight [12, 18, 20, 22–24, 26, 27] also assessed the effect of smoking on the relation of aspirin use and lung cancer risk (Table 1).

**Table 1:** Characteristics of studies included in the meta-analysis of aspirin use and risk of lung cancer.

Author	Year	Country	Study Design	Sex	Cases	Cohorts/Controls	Exposure Definition	RRs/ORs	Adjustments
Cook NR	2013	USA	RCT^∗^	F	431	39876	100mg alternate day	1.04 (0.86–1.26)	None
Lim WY	2012	Singapore	C, H	F	257	561	>2 times/Wk, > = 1 month	0.50 (0.31–0.81)	1–6
McCormack VA	2011	Mixed	^£^7 C+1 Cohort	F	4309	58301	Any aspirin vs non-use	1.02 (0.87–1.19)	1,2,8,10,11,15
				M				0.73 (0.57–0.92)	
Slatore CG	2009	USA	Cohort	F+M	665	77125	Average^Ф^>3d/Wk in 10 y	0.90 (0.71–1.15)	1,7,10,11,14
Van Dyke AL	2008	USA	C, P	F	580	541	> = 325mg*3/Wk, >1month	0.66 (0.46–0.94)	1,2,8–12
Olsen JH	2008	USA	C	F+M	573	857	>4prescriptions/y^Я^	0.75 (0.49–1.14)	1,2,7,10,14,16
Kelly JP	2008	USA	C, H	F+M	1884	6251	> = 4d/Wk, >5y	1.10 (0.90–1.20)	1,2,7,10,11,14,17–19
Jacobs EJ	2007	USA	Cohort	F+M	1815	146113	> = 325mg/d, > = 5y	0.98 (0.76–1.25)	1,2,7–10,13,20–25
Hernández-Díaz S	2007	USA	Nested-C	F+M	4336	14336	> = 150mg/d, >1 y	1.53 (1.22–1.92)	1,7,9,10,12–14,19,25,26
Harris RE	2007	USA	C, P	F+M	492	984	>325mg *3/Wk, > = 5y	0.36 (0.22–0.58)	1,6–9,11,13,19
Feskanich D	2007	USA	Cohort	F	1360	109348	3-5tables/Wk, >2 y	0.98 (0.79–1.21)	1,10,11
Hayes JH	2006	USA	Cohort	F	403	27162	2-5tables/Wk	0.85 (0.60–1.19)	1–3,9–11,13,19
Muscat JE	2003	USA	C, H	F+M	1038	1002	1-5tables/Wk, > = 1y	0.65 (0.45–0.93)	1,2,7,11
Holick CN	2003	USA	Cohort	M	328	49383	> = 2 times/Wk	0.89 (0.47–1.67)	1,10
Moysich KB	2002	USA	C, H	F+M	868	935	1 times/Wk, >1y	0.57 (0.41–0.78)	1,2,10
				F				0.52 (0.29–0.95)	
				M				0.62 (0.43–0.90)	
Akhmedkhanov A	2002	USA	Nested-C	F	81	808	> = 3times/Wk, > = 6months vs non-use	0.66 (0.34 0 1.28)	2,10
Schreinemachers DM	1994	USA	Cohort	F+M	163	12668	Any use in 1 month vs non-use	0.68 (0.49–0.94)	1,7
Paganini-hill A	1989	USA	Cohort	F+M	111	13869	Daily aspirin use	0.92 (0.54–1.55)	None
				F				0.27 (0.07–1.13)	
				M				1.30 (0.72–2.35)	

^∗^: Represents ‘Post-trial of Randomized Controlled Trial’.

^£^: McCormack VA et.al investigated 8 studies from ILCCO that had data on aspirin or NSAID use prior to diagnosis, including 7 case-control studies and 1 cohort study.

^Ф^: In this cohort, ‘total average use over the 10 years’ was estimated by multiplying usual days per week by the number of years, using the midpoints of the categories, divided by 10.

^Я^: Reference defined as “no self-reported use or prescriptions more than 1 year before index date”.

Adjustment: 1. Age, 2. Education, 3. Fruit consumption, 4. Vegetable consumption, 5. Housing type, 6. History of cancer in 1^st^ degree relative, 7. Sex, 8. Race, 9. Body mass index, 10. Smoking status, 11. Pack-years, 12. History of chronic obstructive pulmonary disease, 13. History of ulcer, migraine or chronic headache, osteoarthritis or chronic joint pain, rheumatoid arthritis, coronary artery disease, 14. Use of other NSAIDs, 15. Year of birth, 16. Study, 17. Study region, 18. Interview year, 19. Alcohol use, 20. Physical activity level, 21. Use of hormone replacement therapy, 22. History of mammography or colorectal endoscopy, 23. History of PSA testing, 24. Diabetes, 25. Hypertension, 26. Calendar year.

Abbreviation: VITAL: VITamins And Lifestyle cohort, RCT: Randomized controlled study, C: case-control study, H: Hospital-based, P: Population-based, d: day, Wk: week, y: year, vs: versus, F: female, M: male, RR: relative risk, OR: odds ratio, ILCCO: International Lung Cancer Consortium, SCLC: Small cell lung cancer, CPS II NC: Cancer Prevention Study II Nutrition Cohort, IWHS: Iowa Women’s Health Study, HPFS: The Health Professionals Follow-Up Study, NHEFS: NHANES I Epidemiologic Follow-up Studies, NHANES: National Health and Nutrition Examination Survey.

**Fig 2:**
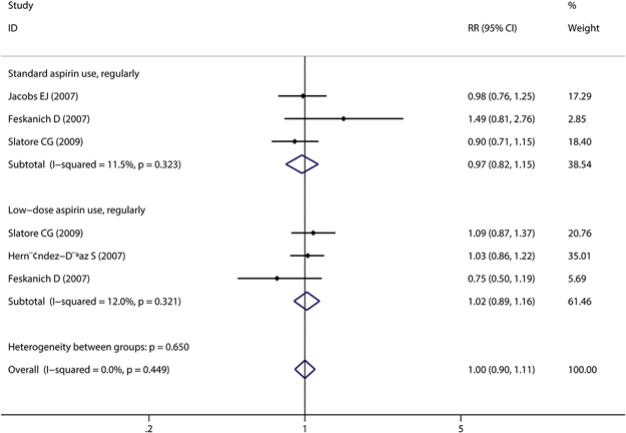
Forest plot and meta-analysis of regular aspirin use and incidence of lung cancer, stratified by dose of usage. Standard aspirin use refers to “regular aspirin use with dose ≥325mg”, while low-dose aspirin use is considered for “regular aspirin use with dose between 75 to 100mg”. The solid diamonds and horizontal lines correspond to the study-specific estimated risks and 95% CIs. Besides, the hollow diamonds represent the pooled relative risk and 95% CIs. Abbreviation: RR: relative risk, CI: confidence interval.

**Fig 3:**
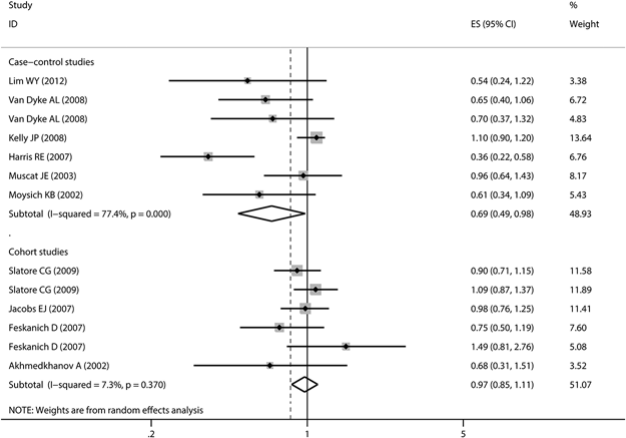
Forest plot and meta-analysis of long time regular aspirin use and lung cancer risk among cohort studies, stratified by study design. ES refers to relative risk among cohort studies and odds ratio among case-control studies. Long time regular aspirin use was defined as “regular aspirin use for more than 5 years, regardless of the dose”. The solid diamonds and horizontal lines correspond to the study-specific estimated risks and 95% CIs. Besides, the hollow diamonds represent the pooled relative risk and 95% CIs. Abbreviation: CI: confidence interval.

**Fig 4:**
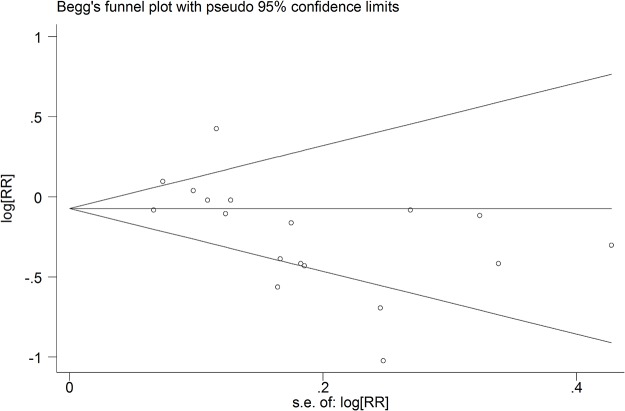
Begg’s funnel plot of studies on aspirin use and risk of lung cancer for evaluation of publication bias.

Any aspirin use was associated with a decreased risk of lung cancer in case-control studies with a large evidence of heterogeneity (pooled OR: 0.71, 95%CI: 0.56–0.91; I^2^: 82.3%). However, no association was detected in cohort studies (pooled RR: 0.97, 95%CI: 0.81–1.15). There was also some evidence of heterogeneity, which could be subsided by excluding the two studies[29, 30] that did not adjust for smoking (I^2^ = 55.2%, I^2^ = 10.3%, respectively) (detailed estimated data are shown in Table 2 and Table 3). As for regular standard aspirin use, similar insignificant results were obtained in cohort studies (pooled RR: 0.97, 95%CI: 0.82–1.15) (Fig 2). However, in case-control studies, a stronger inverse association was detected (pooled OR: 0.61, 95%CI: 0.42–0.89). For low-dose regular aspirin use, both groups did not yield any significant associations, which confirmed the data abstracted from a post-trial of RCT [12]. In this large-scale study, 33682 women were included; after over ten years of follow-up, no significant association was found between the 100mg alternate-day aspirin use group and placebo patients (HR: 1.04, 95%CI: 0.86–1.26). Some of the included studies[7, 8, 10, 18, 20, 22–25, 28] reported the estimated data about long time aspirin use and lung cancer risk. The pooled estimates revealed similar results as regular aspirin use (Fig 3). Furthermore, we conducted a subgroup analysis based on gender and found that males may benefit more from aspirin use than females (pooled RR for men: 0.83, 95%CI: 0.48–1.41; pooled RR for females: 1.02, 95%CI: 0.70–1.47). However, statistical significance level was not reached. After stratification by pathological type, we found that aspirin might protect more people suffering from NSCLC (pooled RR: 0.67, 95%CI: 0.25–1.86). Finally, obvious publication bias was found by Egger’s (P = 0.015) and Begg’s (P = 0.028) tests (Fig 4). Subsequently, sensitivity analyses indicated that no single study influenced the pooled RR qualitatively.

**Table 2:** Summary risk estimates of the association between aspirin use and the lung cancer incidence among cohort studies.

Outcomes of interest	Num	RR and it’s 95%CI	p value		Heterogeneity		Model
				Q-statistic	I^2^ Value	p value	
Cohort studies	9	0.97 (0.81–1.15)	0.690	22.31	55.2%	0.014	Random
Cohort studies^∮^	7	1.05 (0.95–1.16)	0.348	6.69	10.3%	0.351	Fixed
Gender							
Men only	3	0.83 (0.48–1.41)	0.485	6.17	67.6%	0.046	Random
Women only	5	1.02 (0.70–1.47)	0.937	7.61	47.4%	0.107	Random
Regular standard aspirin use (> = 325mg)	3	0.97 (0.82–1.15)	0.725	2.26	11.5%	0.323	Fixed
Low-dose aspirin use (75–100mg)	3	1.02 (0.89–1.16)	0.290	2.27	12%	0.321	Fixed
Long-term aspirin use (> = 5 years)	4	0.98 (0.86–1.11)	0.700	5.39	7.3%	0.370	Fixed
Long-term tandard aspirin use (> = 5 years)	3	0.97 (0.82–1.15)	0.725	2.26	11.5%	0.323	Fixed
Long-term low-dose aspirin use(> = 5 years)	2	0.95 (0.67–1.35)	0.768	2.24	55.4%	0.134	Random
Long-term any dose aspirin use (> = 5 years)	1	0.68 (0.31–1.51)	0.340	NA	NA	NA	NA
Pathological type							
NSCLC	2	0.67 (0.25–1.86)	0.447	3.01	66.8%	0.083	Random
Adenocarcinoma (female only)	1	1.50 (0.92–2.45)	NA	NA	NA	NA	NA
Squamous cell carcinoma (female only)	1	1.31 (0.68–2.51)	NA	NA	NA	NA	NA
SCLC (female only)	1	1.46 (0.68–3.13)	NA	NA	NA	NA	NA

^∮^: Excluded two studies which did not adjusted for smoking status.

Abbreviation: RR: relative risk, CI: confidence interval, Num: number, NSCLC: non-small cell lung cancer, SCLC: small cell lung cancer, NA: not applicable.

**Table 3:** Summary risk estimates of the association between aspirin use and the lung cancer incidence among case-control studies.

Outcomes of interest	Num	OR and it’s 95%CI	p value		Heterogeneity		Model
				Q-statistic	I^2^ Value	p value	
Case-control studies	8	0.71 (0.56–0.91)	0.007	39.62	82.3%	0.000	Random
Study type							
Hospital-based case-control studies	4	0.75 (0.49–1.14)	0.177	23.27	87.1%	0.000	Random
Population-based case-control studies	4	0.65 (0.42–1.01)	0.054	15.01	80.0%	0.002	Random
Gender							
Men only	2	0.70 (0.57–0.85)	0.000	0.53	0.0%	0.467	Fixed
Women only	4	0.67 (0.44–1.02)	0.060	16.79	82.1%	0.001	Random
Standard aspirin use, regularly (> = 325mg)	4	0.61 (0.42–0.89)	0.010	9.55	68.3%	0.023	Random
Low-dose aspirin use, regularly (75–100mg)	1	0.70 (0.37–1.32)	NA	NA	NA	NA	NA
Long-term aspirin use (> = 5 years)	6	0.69 (0.49–0.98)	0.038	26.56	77.4%	0.000	Random
Long-term tandard aspirin use (> = 5 years)	3	0.62 (0.35–1.09)	0.094	9.32	78.5%	0.009	Random
Long-term low-dose aspirin use(> = 5 years)	1	0.70 (0.37–1.32)	0.272	NA	NA	NA	NA
Long-term any dose aspirin use (> = 5 years)	3	0.79 (0.47–1.31)	0.358	6.3	68.3%	0.043	Random
Pathological type							
NSCLC	3	0.76 (0.56–1.02)	0.071	7.22	72.3%	0.027	Random
Adenocarcinoma (female only)	4	0.94 (0.84–1.05)	0.253	2.74	0.0%	0.434	Fixed
Squamous cell carcinoma (female only)	3	0.93 (0.80–1.09)	0.386	1.35	0.0%	0.509	Fixed
SCLC (female only)	4	0.75 (0.48–1.17)	0.202	10.25	70.7%	0.017	Random

Abbreviation: OR: odds ratio, CI: confidence interval, Num: number, NSCLC: non-small cell lung cancer, SCLC: small cell lung cancer, NA: not applicable.

## Discussion

The findings of the present meta-analysis showed that there is no explicit association between aspirin use and lung cancer risk among cohort studies, which was consistent with previous meta-analyses [15–17]. Among the previous meta-analyses, Bosetti C et.al [17] aimed at extensively explaining the association between aspirin use and risk of 12 selected cancer, without further exploring the influence of aspirin use on lung cancer risk. Besides, another two studies [15, 16] also tried to investigate the possible relations, taking the dose-risk and duration-risk relationships into consideration. But neither of them presented meta-analyses stratified by study design throughout the manuscript. In this meta-analysis, we found that statistical significance was mostly contributed by case-control studies. A spurious protective effect may be presented due to its nature of selection and recall bias. Four of seven case-control studies in our meta-analysis were hospital-based, which might more easily introduce bias. It is known that cohort studies are less susceptible to bias due to their prospective design. So the results summarized in cohort studies are more credible and stable. To minimize the influence of study design, we performed this meta-analysis separately assessing cohort- and case-control studies. Besides, all these studies were based on electronic database before September 2011. After that, several studies [12, 18] were published which, in some extend, might provide more evidence on these associations. Thus we performed this meta-analysis to further understand whether the aspirin use could influence the risk of lung cancer.

When we limited our analysis to long-term regular aspirin use, a strong inverse association was reported in case-control studies (pooled OR: 0.69, 95%CI: 0.49–0.98), but not for cohort studies (pooled RR: 0.98, 95%CI: 0.86–1.11). Thorat et al.[31] claimed that growing evidence supports aspirin’s effect in reducing cancer incidence and mortality, but the duration of use needs to be at least 5 years. In our analysis, after we subdivided regular aspirin use into standard and low-doses, an insignificant difference was obtained between these two subgroups among cohort studies (pooled RR = 0.97, pooled RR = 0.95, respectively). But in case-control studies, slightly stronger insignificant inverse association was detected among individuals with standard aspirin use compared with those taking low-dose aspirin (pooled RR = 0.62, pooled RR = 0.70, respectively). A recently published population-based cohort study [14]indicated that low-dose aspirin in the year prior to diagnosis was associated with lower tumor extent and fewer metastatic diseases in lung cancer, suggesting a protective effect of low-dose aspirin use on lung cancer. Taken together, these data suggest that long-term aspirin use might reduce the incidence of lung cancer; however, the appropriate dose needed is still unclear.

Smoking is considered the most well-known environmental risk factor for lung cancer[32]. Almost all the included studies were adjusted for smoking status or pack-years except two reports[29]. After exclusion of both studies, the estimated data only slightly changed, maybe because actual pack-years and smoking status are hard to adjust. However, it was impossible to conduct an analysis on aspirin use and risk of lung cancer stratified by smoking pack-years/status due to the different measurements used in various studies. Three of the eight studies [18, 23, 24]demonstrated that aspirin use was associated with a reduced risk of lung cancer among smokers. Another study [26]reported that estimated RR in male smokers was higher than in non-smokers (RR = 1.08, RR = 0.96, respectively), but the difference was not statistically significant.

In cohort studies, a stronger insignificantly inverse association between aspirin use and lung cancer was reported in male populations than in females (pooled RRs of 0.83 and 1.02 for men and women, respectively). A research conducted by Chen et al.[33]found that the levels of serum E2, prolactin and follicle-stimulating hormone were remarkably increased in male patients with lung cancer, suggesting that sexual hormones may play a role in lung cancer genesis. Several other studies suggested that estrogen receptors may influence the effect of aspirin use on lung cancer [16, 34]. Further studies are needed to clarify the influence of sex on the association between aspirin use and lung cancer risk.

Several limitations of the present meta-analysis should be mentioned. Firstly, approximately half of the included studies were case-control trials. Although they were not combined in this meta-analysis, there are still several potential confounding factors, which may influence the conclusion obtained from these studies. Secondly, the included studies were different in terms of populations, doses, durations of aspirin use, selection of controls and confounders adjusted, all of which may affect the possible association between aspirin use and lung cancer risk. Subsequently, subgroup- and sensitivity analyses were performed. However, these analyses were far from removing all heterogeneity. Thirdly, our literature search was restricted to published studies. It is known that negative studies are less likely to be published in indexed journals, which may bias our results. Finally, due to lack of relevant data, it was impossible to clarify the dose-response association. Therefore, it was hard to quantitatively assess the aspirin use on lung cancer risk. Taken together, prospective studies with different dose of aspirin use, especially RCTs, were needed to solve this question.

## Conclusion

In summary, our meta-analysis indicated that regular/any aspirin use might have no association with lung cancer risk based on findings of cohort studies. Long time aspirin use, sex, dose and type of lung cancer might alter the effect of aspirin use on lung cancer risk. Further well-designed large-scale studies are needed to confirm our findings.

## Supporting Information

S1 PRISMA Checklist (DOC)
